# Myxovirus resistance 1 (MX1) is an independent predictor of poor outcome in invasive breast cancer

**DOI:** 10.1007/s10549-020-05646-x

**Published:** 2020-04-29

**Authors:** Abrar I. Aljohani, Chitra Joseph, Sasagu Kurozumi, Omar J. Mohammed, Islam M. Miligy, Andrew R. Green, Emad A. Rakha

**Affiliations:** 1 Nottingham Breast Cancer Research Centre, Division of Cancer and Stem Cells, School of Medicine, University of Nottingham Biodiscovery Institute, University Park, Nottingham, NG7 2RD UK; 2 Department of Clinical Laboratory Sciences, Faculty of Applied Medical Sciences, Taif University, Taif, Saudi Arabia; 3 School of Medicine, Nottingham City Hospital, University of Nottingham, Nottingham, UK; 4 Histopathology Department, Faculty of Medicine, Menoufia University, Shibïn al-Kawm, Egypt; 5 Department of Histopathology, Nottingham University Hospital NHS Trust, City Hospital Campus, Hucknall Road, Nottingham, NG5 1PB UK

**Keywords:** Myxovirus resistance 1, MX1, Breast cancer, Progression, Prognosis

## Abstract

**Background:**

Breast cancer (BC) is a disease with variable morphology, clinical behaviour and response to therapy. Identifying factors associated with the progression of early-stage BC can help understand the risk of metastasis and guide treatment decisions. Myxovirus resistance 1 (MX1), which is involved in the cellular antiviral mechanism, plays a role in some solid tumours; however, its role in invasive BC remains unknown. In this study, we aimed to explore the clinicopathological and prognostic significance of MX1 in BC.

**Methods:**

MX1 was assessed at the protein level using tissue microarrays from a large well-annotated BC cohort (*n* = 845). The expression of *MX1* mRNA was assessed at the transcriptomic level using the Molecular Taxonomy of Breast Cancer International Consortium (METABRIC; *n* = 1980) and validated using three publicly available cohorts on Breast Cancer Gene-Expression Miner (bc-GenExMiner version 4.4). The associations between MX1 expression and clinicopathological factors, and outcome were evaluated.

**Results:**

High MX1 protein expression was associated with features of aggressiveness, including large tumour size, high tumour grade, high Nottingham prognostic index scores, hormone receptor negativity and high Ki67 expression. High MX1 expression showed an association with poor patient outcome and it was an independent predictor of short BC-specific survival (*p* = 0.028; HR = 1.5; 95% CI = 1.0–2.2). Consistent with the protein results, high *MX1* mRNA levels showed an association with features of aggressive behaviour and with shorter survival.

**Conclusion:**

This study identified MX1 as an independent predictor of poor outcome in patients with BC. Further functional studies are needed to investigate the biological role of MX1 in BC and its potential value as a therapeutic target.

**Electronic supplementary material:**

The online version of this article (10.1007/s10549-020-05646-x) contains supplementary material, which is available to authorized users.

## Introduction

Breast cancer (BC) comprises different diseases that show distinct molecular features, clinical behaviour and response to therapy. Therefore understanding BC biology and defining a personalised therapy method remains a challenge [1]. Deciphering the molecular mechanisms and biological processes controlling BC progression is important to develop better treatment strategies and ultimately improve patient outcome.

One of the interferon‐induced GTPases that belongs to the dynamin superfamily of large GTPases is MX1 protein, also known as MXA, which is encoded by Myxovirus resistance 1 (*MX1*) gene [2]. Previous studies have indicated that MX1 has many features, including a tendency to self‐assemble, low affinity for guanosine triphosphate (GTP) and high intrinsic rate of GTP hydrolysis. MX1 is mainly localised in the cytoplasm and shows a granular staining pattern that may be associated with cytoskeletal structures [3]. MX1 releases GTP, which plays a role in metabolising the protein that contributes to the cellular antiviral mechanism [4]. MX1 is overexpressed and appeared to play a role in a variety of cancers but its effect remains controversial. Many interferon-stimulated genes (ISGs) can inhibit the motility of the transformed cells and the invasion of normal tissues. In most tissues, MX is highly induced by the ISG, particularly interferon (IFN) α and β. MX1 also has a role in the inhibition of motility and invasiveness in some cancers, such as prostate carcinoma and melanoma [5, 6]. However, MX1 appears to have different effects in other cancers. It was observed that reduced MX1 level can suppress apoptosis during cancer development [6]. In colorectal cancer, MX1 protein is overexpressed and plays a significant role in lymph node metastasis. An in vitro study conducted in colorectal carcinoma indicated that knockdown of *MX1* in colon cancer cells inhibits migration and invasion of tumour cells [7]. Overexpression of MX1 in BC has been reported previously in certain IHC subtype, highlighting its correlation with immune response and tumour infiltrating lymphocytes, TILs [8] and it has been associated with anthracycline-based chemotherapy response [9].

MX1 responds to type-1 IFN and acts as a mediated signalling pathway [10]. The decrease in MX1 leads to the imperfection of lymphocytes of early and advanced stages of BC which is a consequence of IFN-α signalling in T and B cells [11]. According to Han et al. [11], IFN-α signalling defects in lymphocytes of early and advanced staged BC is associated with a decrease MX1 level. Type-1-IFN influences tumour reduction and development by acting on tumour, immune, or even endothelial cells [9] and can hinder angiogenesis through vascular endothelial growth fact (VEGF) down-regulation [10]. However, the prognostic value of MX1 in BC remains to be defined. The aim of this study is to investigate the expression of MX1 in early-stage (operable) BC and assess its association with clinicopathological parameters and patient outcome as a potential prognostic factor and a possible therapeutic target in BC.

## Materials and methods

### Study cohorts

A large well-characterised early-stage primary operable invasive BC cohort from female patients attended at Nottingham City Hospital, Nottingham, UK, between 1998 and 2006 was used in this study as described in previous studies [12, 13]. All patients were aged less than or equal to 70 years and were treated as per a uniform protocol. Clinicopathological data were systematically recorded, including patient age, menopausal status, tumour grade, tumour size and histological type. None of the patients in this study was offered neoadjuvant therapy. During the time of the study cohort presentation, patients were treated based on the Nottingham local protocol, which was based on the Nottingham prognostic index (NPI) and ER status as previously published [14]. Briefly, patients with good prognostic NPI scores (≤ 3.4) were not prescribed adjuvant chemotherapy. Patients with higher NPI scores were treated with adjuvant chemotherapy if they have ER-negative tumours. ER-positive patients were treated with hormone therapy.

Hormonal receptor status including oestrogen receptor (ER) and progesterone receptor (PgR) was available and the positive status was defined as those tumours with ≥ 1% immunoreactivity [15, 16]. HER2 and Ki67 status were also available. Ki67 positivity was considered when > 10% of the tumour cells are positive. The assessment of HER2 status was carried out using immunohistochemistry and a chromogenic in situ hybridisation technique to evaluate the gene amplification for the cases with borderline (+ 2). The definition for HER2 positivity was ≥ 10% of tumour cells showing intense staining of their membranous (score + 3) [15, 17, 18]. Based on the immunohistochemistry (IHC) profile, BC molecular subtype data were used, including luminal A, luminal B, HER2+ and triple negative (TN) defined as (Ki67 < 10% (low proliferation); ER+/HER2−), (Ki67 ≥ 10% (high proliferation); ER+/HER2−), (HER2+ irrespective of ER) and (ER−, PgR− and HER2−), respectively [19]. To further understand the molecular interactions of these biomarkers, basal cytokeratin (CK5, CK17 and EGFR), proliferation marker and epithelial mesenchymal transition (EMT)-associated markers, comprising E-cadherin and N-cadherin, were used [20, 21]. Follow-up data were recorded from the date of the primary surgery to the time of death due to BC, which is defined as BC-specific survival (BCSS) and the time from surgery until developing distant metastasis, which is defined as distant-metastasis-free survival (DMFS).

### MX1 protein expression

#### Western blot (WB) for antibody specificity validation

Using WB, the primary antibody, rabbit polyclonal anti-MX1 antibody (ab95926, Abcam, UK), was validated. BC cell line lysate, MCF7, and human embryonic kidney (HEK) that was used as a control (from the American Type Culture Collection, Rockville, MD, USA) were employed for WB antibody specificity validation. MX1 antibody was used at a dilution of 1:1500 and IRDye 800CW Donkey anti-Rabbit fluorescent secondary antibody (LI-COR Biosciences) was used at a 1:15,000 dilution. For loading control, mouse monoclonal anti-β-actin primary antibody (1:5000, Sigma-Aldrich) was used and followed by incubation with anti-Mouse fluorescent secondary antibody (LI-COR Biosciences). To detect the protein molecular weight, 20 µg of the cell lysate was loaded alongside the protein ladder (Page Ruler Plus Prestained Protein Ladder, Thermo Scientific). A specific band was detected at the predicted molecular weight of ~ 64 kDa using Odyssey Fc scanner and visualised by Image Studio 4.0 software (Supplementary Fig. 1).

#### Immunohistochemistry (IHC)

To assess the pattern of MX1 expression, a representative full-face tissue section for invasive BC (*n* = 10) for different molecular BC subtypes, histological types and tumour grade were stained to verify the staining homogeneity before the staining of tissue microarray (TMA). Tumour samples were arrayed as previously prepared as TMA utilising a TMA Grand Master® (3DHISTECH®, Budapest, Hungary) [13, 22]. According to the manufacturer’s recommendations, antigen retrieval using citrate buffer pH 6.0 at 1000 W for 20 min with microwave energy was performed. The MX1 protein expression was evaluated by IHC utilising the Novocastra Novolink™ Polymer Detection Systems kit (Leica, Biosystems, UK). Briefly, tissue sections (4 µm) were incubated for 60 min with rabbit polyclonal MX1 (dilution 1:100). A positive control using normal kidney tissue was used, whereas omitting the primary antibody was used as a negative control.

#### Scoring of MX1 expression

The evaluation of MX1 cytoplasmic expression was performed utilising a modified histochemical score (*H*-score) for the semi-quantitative analyses of immunoreactivity [23]. To produce a range of values between 0 and 300, the staining intensity [(0 (negative), 1 (weak), 2 (moderate), 3 (strong)] multiplied by the percentage (0–100%) for each intensity of representative cells in the tissue. All non-informative cores were excluded from the scoring including cores with only normal breast tissue or folded tissues during processing and cores contain < 15% tumour cells. To calculate inter-observer concordance, double scoring was blindly performed by AA with (~ 10%) scored by another scorer (IM). Based on BCSS, X-tile bioinformatics software version 3.6.1 (Yale University, USA) was used to generate cut-off points to dichotomise MX1 protein into high and low expression using an *H*-score of 110.

### *MX1* transcriptomic analysis

The Molecular Taxonomy of Breast Cancer International Consortium (METABRIC) (*n* = 1980) was utilised to assess *MX1* mRNA expression [24]. Based on the median, a cut-off to dichotomise the levels of mRNA expression into low and high subgroups was employed. The association between *MX1* mRNA level, clinicopathological factors and patient outcome was evaluated. Three more publicly available datasets (bc-GenExMiner) version 4.4 (https://bcgenex.centregauducheau.fr) as a prognostic analytical module were also employed in this study to validate the data of METABRIC cohort (*n* = 1980), namely, DNA microarrays Affymetrix (*n* = 4904), RNA-Seq TCGA (*n* = 1034) and RNA-Seq GSE81540 (*n* = 3678).

### Statistical analysis

SPSS® Statistics 24.0 was utilised for the statistical analysis (SPSS, Inc., Chicago, IL, USA). To evaluate the concordance rate between both scorers, the interclass correlation coefficient (ICC) statistical test was performed. The relationship between the targets and the clinicopathological factors was determined using a *χ*^2^ test. The correlation between the transcriptome and protein levels was assessed by Spearman’s rank correlation coefficient. For the univariate survival analysis, log-rank test and Kaplan–Meier curves were used. Cox regression model including other prognostic co-variables (tumour grade, nodal stage, tumour size, HER2 status and basal phenotype) was used to detect the independent prognostic value of MX1. For the whole analysis, *p*-value of < 0.05 was considered significant.

## Results

### Patterns of MX1 protein expression

**Fig. 1:**
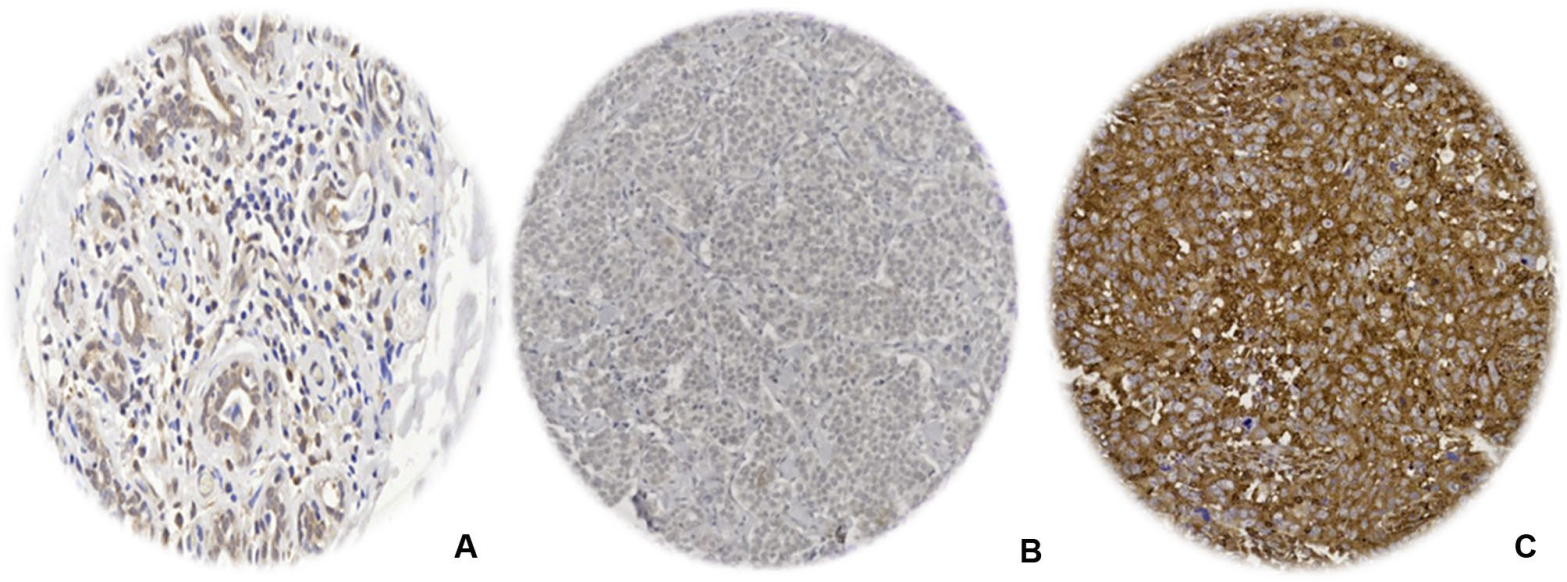
Photomicrographic images (× 40) for immunohistochemical protein expression of MX1 in breast tissue microarray images; MX1 expression of the cytoplasm in cancer cells was distributed as follows; **a** normal breast terminal duct-lobular, **b** negative expression in invasive breast carcinoma, and **c** positive expression in invasive breast carcinoma (high expression is attributed to *H*-score cut-off point of 110 or more based on X-tile for dichotomisation)

Normal breast terminal ductal lobular units displayed a weak MX1 cytoplasmic staining. In the tumour cells, when present, MX1 was expressed in the cytoplasm with no discernible membranous or nuclear staining observed (Fig. 1).

After the exclusion of non-informative TMA cores, the total number of cases suitable for the scoring was 845 out of 2000 cases. A strong concordance was found between both scorers in MX1 scoring (ICC = 0.959, *p* < 0.0001). A total of 243/845 (28%) of the BC cases showed high expression of MX1 protein.

### Significance of MX1 protein expression

High MX1 protein expression was significantly associated with a large tumour size (*p* = 0.011), high histological grade, poor NPI, hormonal receptor negativity (ER− and PR−) (all *p* < 0.0001), younger age at diagnosis (*p* = 0.047) and premenopausal status (*p* = 0.040, Table 1). Based on St. Gallen International Expert Guidelines and by using the available data in IBC cohorts, high protein expression of MX1 was significantly associated with the triple negative subtype (*p* < 0.0001, Table 2).

**Table 1:** Associations between MX1 protein expression and the clinicopathological factors in breast cancer (*n* = 845)

Clinicopathological parameters	MX1 expression		*χ*^2^ (*p*-value)
	Low (*N* = 602)	High (*N* = 243)	
	*N* (%)	*N* (%)	
Age (years)			
< 50	222 (68)	105 (32)	3.938 **(0.047)**
≥ 50	423 (74)	148 (26)	
Menopausal status			
Pre-menopausal	237 (68)	113 (32)	6.431 **(0.040)**
Post-menopausal	406 (75)	137 (25)	
Peri-menopausal	3 (100)	0 (0)	
Tumour size (cm)			
< 2	338 (76)	109 (24)	6.497 **(0.011)**
≥ 2	303 (68)	143 (32)	
Tumour grade			
Low	129 (91)	13 (9)	52.618 **(< 0.0001)**
Moderate	212 (79)	57 (21)	
High	302 (62)	181 (38)	
Nodal stage			
1	401 (72)	158 (28)	0.224 (0.894)
2	188 (72)	74 (28)	
3	55 (74)	19 (25)	
Nottingham prognostic index			
Poor	95 (64)	53 (36)	25.829 **(< 0.0001)**
Moderate	328 (68)	156 (32)	
Good	218 (84)	43 (16)	
Lymph-vascular invasion			
Negative	426 (73)	161 (27)	0.620 (0.431)
Positive	213 (70)	91 (30)	
Oestrogen receptor			
Negative	132 (56)	195 (44)	41.725 **(< 0.0001)**
Positive	512 (78)	147 (22)	
Progesterone receptor			
Negative	234 (62)	143 (38)	32.813 **(< 0.0001)**
Positive	398 (80)	102 (20)	
HER2 status			
Negative	538 (71)	216 (29)	0.431 (0.511)
Positive	84 (74)	29 (26)	
Basal phenotype			
Negative	499 (75)	170 (25)	10.583 **(0.001)**
Positive	133 (63)	78 (37)	
Ki67			
Low	228 (84)	43 (16)	27.456 **(< 0.0001)**
High	296 (66)	151 (34)	

Significant *p* values are in bold

**Table 2:** The association between MX1 protein expression and different IHC subtypes

IHC subtypes	MX1 expression		*χ*^2^ (*p*-value)
	Low (*N* = 549) *N* (%)	High (*N* = 224) *N* (%)	
Luminal A	172 (85)	31 (15)	67.991 **(< 0.0001)**
Luminal B	214 (74)	74 (26)	
HER2 enriched	83 (74)	29 (26)	
Triple negative	80 (47)	90 (53)	

Significant *p* values are in bold

High MX1 level was also associated with basal-like phenotype as defined by the positivity of CK5 (*p* < 0.0001), CK17 (*p* = 0.002), EGFR (*p* = 0.007) and expression of EMT-related marker E-cadherin negativity (*p* = 0.003). High MX1 level was also associated with the high expression of the proliferation marker Ki67 (*p* < 0.0001, Table 3).

**Table 3:** The association between MX1 protein expression and basal and epithelial mesenchymal transition (EMT) biomarkers

Biomarkers	MX1 expression		*χ*^2^ (*p*-value)
	Low (*N* = 648)	High (*N* = 253)	
	*N* (%)	*N* (%)	
CK5			
Negative	411 (73)	153 (27)	25.563 **(< 0.0001)**
Positive	58 (49)	60 (51)	
CK17			
Negative	391 (73)	144 (27)	9.553 **(0.002)**
Positive	52 (57)	39 (43)	
EGFR			
Negative	519 (74)	183 (26)	7.401 **(0.007)**
Positive	114 (64)	65 (36)	
E-cadherin			
Negative	202 (66)	105 (34)	9.077 **(0.003)**
Positive	423 (75)	138 (25)	

Significant *p* values are in bold

### MX1 protein expression and patient outcome

**Fig. 2:**
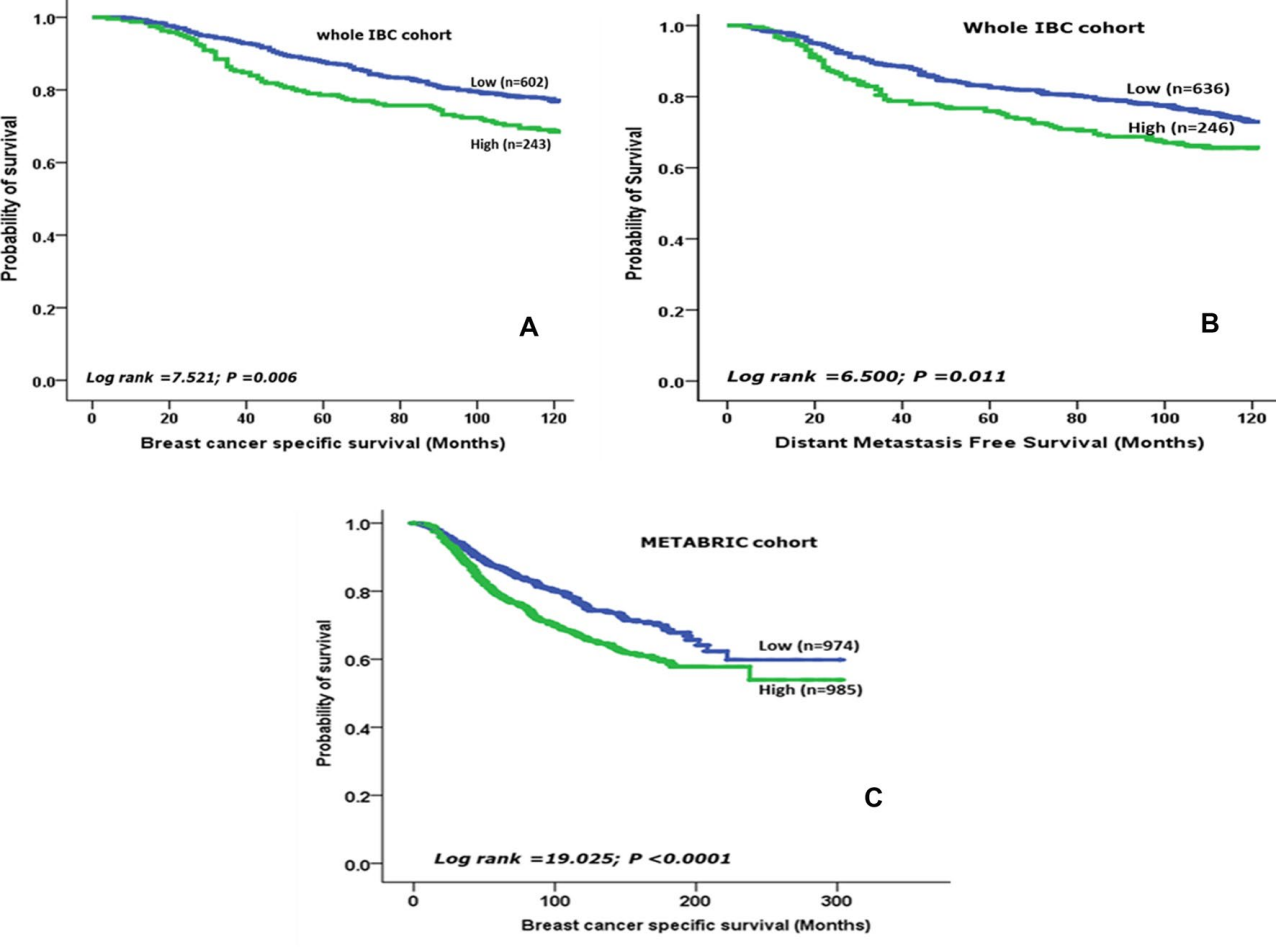
Kaplan–Meier survival plots showing the association between MX1, **a** the protein expression and patient outcome, **b** the protein expression and distant metastasis and **c** mRNA expression (METABRIC) and patient outcome

**Fig. 3:**
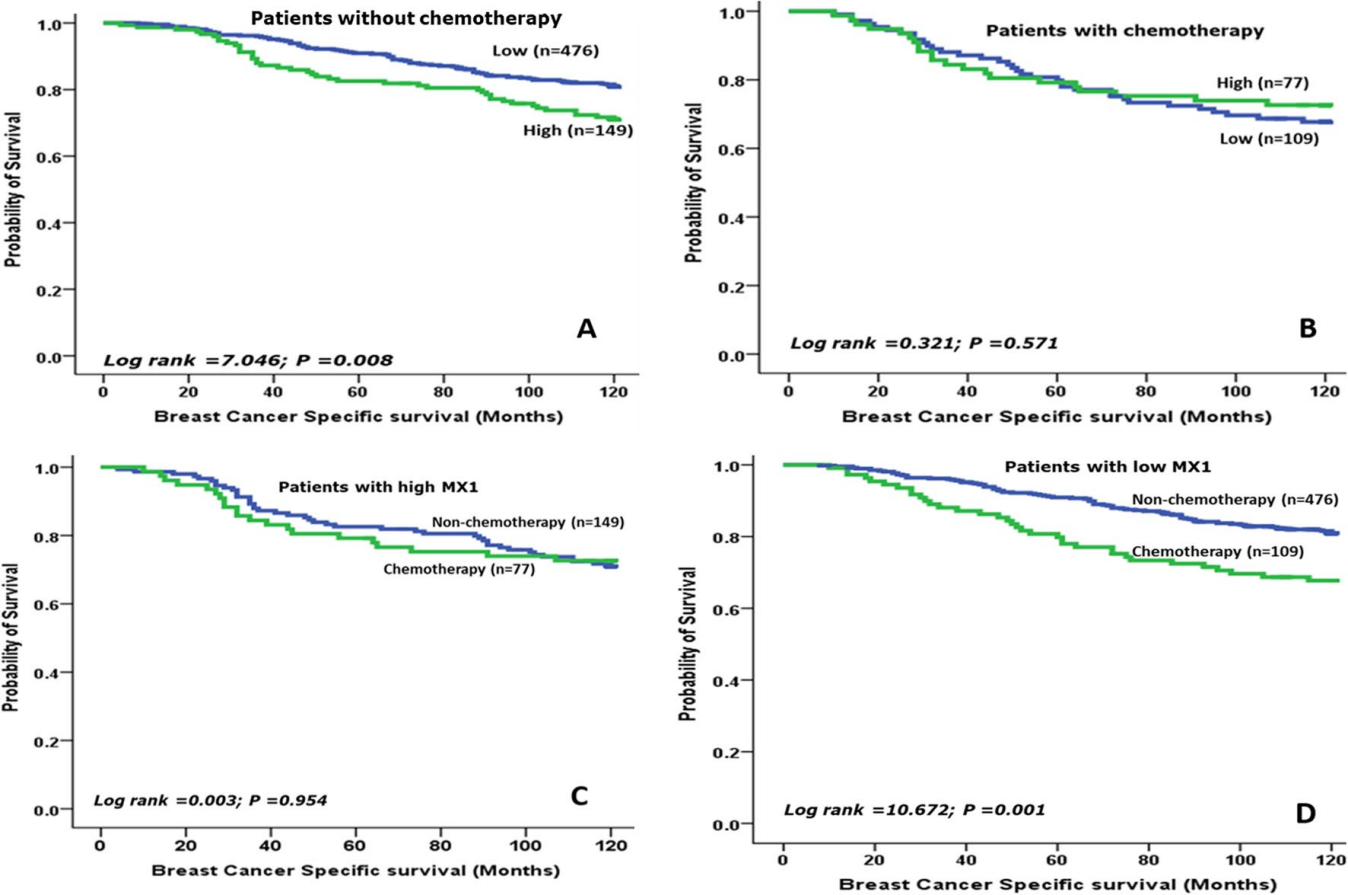
Kaplan–Meier survival plot showing the association between the expression of MX1 protein and breast cancer-specific survival in the invasive breast cancer cohort in **a** patients have not received chemotherapy, **b** patients received chemotherapy, c chemotherapy vs non-chemotherapy classes in patients with high MX1 and **d** chemotherapy vs non-chemotherapy classes in patients with low MX1

High MX1 protein level showed an association with shorter BCSS (*p* = 0.006) and DMFS (*p* = 0.011, Fig. 2a, b). When the cohort was stratified based on adjuvant chemotherapy, high MX1 protein expression was significantly associated with shorter BCSS in patients who did not receive chemotherapy (*p* = 0.008) but lost its prognostic value in those who were offered such therapy (*p* = 0.571, Fig. 3a, b). The cohort was then stratified based on MX1 expression (high- versus low expression subgroups) and the association between adjuvant chemotherapy and outcome was tested. This revealed that in the low MX1 subgroup, chemotherapy was associated with shorter BCSS (*p* = 0.001) whereas in the high MX1 expression subgroup, chemotherapy was not associated with BCSS (*p* = 0.954, Fig. 3c, d).

In multivariate Cox regression analysis, high MX1 protein expression was an independent predictor of shorter BCSS (*p* = 0.028; HR = 1.5; 95% CI = 1.0–2.2) regardless of tumour grade, nodal stage, tumour size, HER2 status and basal phenotype (Table 4). Multivariate Cox regression analysis was performed when the cohort was stratified based on MX1 expression, in low MX1 protein expression cohort, adjuvant chemotherapy was independent predictor of shorter BCSS (*p* = 0.017; HR = 0.493; 95% CI = 0.276–0.879) regardless of, tumour grade, nodal stage, tumour size, HER2 status and basal phenotype (Table 5).

**Table 4:** Multivariate Cox regression for predictors of breast cancer-specific survival and MX1 protein expression in invasive breast cancer

Parameter	Hazard ratio (HR)	95% Confidence interval (CI)		*p*-value
		Lower	Upper	
MX1 protein expression	1.5	1.0	2.2	**0.028**
Tumour grade	3.5	2.1	5.8	** < 0.0001**
Nodal stage	2.3	1.8	2.9	** < 0.0001**
Tumour size	1.5	0.9	2.3	0.056
HER2	2.0	1.3	3.1	**0.001**
Basal phenotype	1.6	1.1	2.3	**0.022**

Significant *p* values are in bold

**Table 5:** Multivariate Cox regression for predictors of breast cancer-specific survival and adjuvant chemotherapy in low MX1 protein expression in invasive breast cancer

Parameter	Hazard ratio (HR)	95% Confidence interval (CI)		*p*-value
		Lower	Upper	
Adjuvant chemotherapy	0.493	0.276	0.879	**0.017**
Tumour grade	3.3	1.5	7.3	**0.003**
Nodal stage	3.0	2.0	4.4	** < 0.0001**
Tumour size	1.0	0.587	1.8	0.942
HER2	1.5	0.781	3.0	0.216
Basal phenotype	1.3	0.708	2.3	0.416

Significant *p* values are in bold

### *MX1* mRNA expression

Based on the spearman’s rank correlation coefficient, a correlation between MX1 protein and *MX1* mRNA expression was observed in the Nottingham subset (*n* = 288) of the METABRIC cohort (*r* = 0.120, *p* = 0.042) indicating weak positive correlation.

Similar to the protein results, high *MX1* mRNA expression was significantly correlated with hormonal status negativity and a high tumour grade (both *p* < 0.0001), positive axillary lymph node (*p* = 0.018), basal-like phenotype (*p* < 0.0001), premenopausal status, younger age (both *p* = 0.001) and LVI positivity (*p* = 0.022, Table 6).

**Table 6:** Association between MX1 mRNA expression and clinicopathological parameters in the METABRIC (*n* = 1980)

Clinicopathological parameters	METABRIC cohort		*χ*^2^ (*p*-value)
	Low (*N* = 990)	High (*N* = 990)	
	*N* (%)	*N* (%)	
Age (years)			
< 50	183 (43)	241 (57)	10.096 **(0.001)**
≥ 50	807 (52)	749 (48)	
Menopausal status			
Pre-menopausal	189 (43)	247 (57)	10.138 **(0.001)**
Post-menopausal	797 (52)	736 (48)	
Tumour size (cm)			
< 2	330 (53)	292 (47)	3.177 (0.075)
≥ 2	652 (49)	686 (51)	
Tumour grade			
Low	118 (69)	52 (31)	103.008 **(< 0.0001)**
Moderate	453 (59)	317 (41)	
High	363 (38)	589 (62)	
Lymph-vascular invasion			
Negative	481 (52)	449 (48)	5.244 **(0.022)**
Positive	291 (46)	344 (54)	
Nodal status			
Negative	544 (53)	491 (47)	5.596 **(0.018)**
Positive	443 (47)	495 (53)	
Oestrogen receptor			
Negative	170 (36)	304 (64)	49.805 **(< 0.0001)**
Positive	820 (54)	686 (46)	
Progesterone receptor			
Negative	395 (42)	545 (58)	45.571 **(< 0.0001)**
Positive	595 (57)	445 (43)	
HER2 status			
Negative	880 (51)	853 (49)	3.372 (0.066)
Positive	110 (44)	137 (56)	
Intrinsic molecular classes			
Luminal A	452 (63)	266 (37)	140.785 **(< 0.0001)**
Luminal B	223 (46)	265 (54)	
HER2 enriched	111 (46)	129 (54)	
Basal like	83 (25)	246 (75)	
Normal like	118 (59)	81 (41)	

Significant *p* values are in bold

In METABRIC, high *MX1* mRNA was significantly associated with poor outcome (*p* < 0.0001, Fig. 2c). Similar results were observed using the bc-GenExMiner version 4.4 for DNA microarray and RNA-Seq cohorts’ analyses. Although no significant difference in the outcome was observed between MX1 high and low expression in TCGA cohort (*n* = 1034, *p* = 0.190, Supplementary Fig. 2), a significant difference between the high and low MX1 expressions in correlation with the probability of patients’ survival was recorded in a DNA microarray cohort (Affymetrix) (*n* = 4904, *p* = 0.0005, Supplementary Fig. 3) either alone or when combined with METBRIC cohort (*n* = 10,001, *p* < 0.0001, Supplementary Fig. 4). Additionally, we have analysed another RNA-Seq cohort, GSE81540 (*n* = 3678), alone or in combination with TCGA and concluded that high MX1 expression was significantly different from low expression and has poorer prognostic consequences in both situations (*p* = 0.0020, 0.0086, Supplementary Figs. 5, 6), respectively. All in all, out of four cohorts analysed and, it was only TCGA cohort, when analysed alone, did not show a significant change between the high and low MX1 expression.

## Discussion

MX1 contributes to the progression of cancers with different attributions, as noticed in various cancers. Although MX1 protein activity craves for antiviral activities in the immunity system [25, 26], it aids in bringing about a signal that articulates a significant contribution to cancer progression and response to treatments, such as chemotherapy procedures. Moreover, the gene replicates and regulates the gene transcription in which alteration is set to take place within the cancer cells. The progression initiates toward the metastatic processes in which cell proliferation, migration and death by the cancer are sustained or inhibited by MX1 [27].

MX1 has been demonstrated to play a role in various human cancers. It has been speculated that it may be a tumour suppressor for IFN therapy [28]. To evaluate the transcriptomic and protein expression level of MX1 by IHC, we used well-annotated multiple BC cohorts to assess their associations with clinicopathological parameters and patient outcomes. The results showed an association between MX1 expression and the clinicopathological features’ characteristic of aggressive behaviour, which strengthens the putative role of MX1 in tumour progression. The weak correlation between the mRNA and protein expression of MX1 may have resulted from different biological and technical factors. One of these biological factors is the rate of mRNA being translated into proteins which is usually termed ‘translational efficiency’ which has been shown to significantly impact on the correlation between mRNA and protein levels [29]. Another reason might be attributed to the subjectivity of *H*-score approach in the interpretation of the expression of IHC-staining sections [30] and the fact that the METABRIC cases used whole tissue comprising many different cell types.

A study has revealed that MX1 is a target for repression of SATB1. Genome organiser SATB1 can promote BC tumour growth and can lead to metastasis by reprogramming SATB1 expression [31]. The pro-proliferative PIK3/AKT pathway plays a role in the regulatory cascade-enhancing MX1 expression in response to IFNα. In relapsing patients, the overexpression of MX1 may be a result of the induction of growth signalling by different pathways [32]. In this study, an association between MX1 grade and the proliferation marker Ki67 is identified.

In different types of cancers, ISGs are largely expressed. IFNs play a significant role in various pathways associated with malignancies. A study revealed that in HER2-positive BCs, MX1 is only expressed in the cytoplasm of tumour cells. Moreover, high histological grade and intense infiltrate of TILs are correlated with the expression of MX1 protein. Type I IFNs lead to T-cell exhaustion by the overexpression of tumour programmed cell death ligand 1 (PD-L1) and the increase of PD-L1 level, which can interact with programmed death 1 on T cells [8].

In this study, high MX1 protein was significantly associated with EGFR and the loss of E-cadherin, which can regulate migration, EMT and invasion [33]. Thus, through EMT, which is an important mechanism for metastasis of breast carcinoma cells, MX1 may play an essential role in the regulation of tumour progression [34].

Additionally, high expression of MX1 was associated with the highly proliferative basal phenotype (CK5 and CK17) [35–37]. To initiate and establish a metastatic cascade, proliferation and invasion must be occurred to the adjacent tissue by the primary tumour cells. At the same time, the evasion of apoptosis and immune responses occurs with the tumour cells [38]. Thus, in cell proliferation, a prerequisite stage of the metastatic process, MX1 may have a role. Furthermore, the elevated level of these basal cytokeratins (CK5 and CK17) in patients with high MX1 expression confers a poor prognosis. Cytokeratins are strongly associated with aggressive behaviours of the tumours such as high histological grade, hormonal receptor negativity and worse outcome [39, 40]. This further supports our results and implies that MX1 plays a role in tumourigenic pathways.

The results indicate that MX1 is a potential prognostic biomarker in IBC particularly in patients not receiving chemotherapy. Interestingly, our analysis of the overall cohort showed that high MX1 level in patients who were not offered chemotherapy was associated with poor outcome in comparison with those who received chemotherapeutic drugs. Consequently, MX1 prognostic value seems to be invalidated when patients were offered chemotherapy. Whether this phenomenon can be exploited to monitor the chemotherapy effectiveness or not remains ambiguous and required further clinical studies to be approved. The MX1 prognostic value has been validated by the publicly available domains. The results demonstrated that high MX1 expression was correlated with shorter survival in BC patients, in three large datasets out of the four cohorts tested. The correlation between MX1 and outcome was also maintained throughout the follow-up period indicating that the impact of MX1 on the outcome is not time dependent.

Although this study presents interesting findings, some hypothetical limitations were determined. Firstly, it is based on a retrospectively collected cohort. Secondly, the cut-off point used in the protein level analysis was not prespecified and was based on X-tile, which was determined based on the prediction of patient survival and that different cut-off points may result in different categorisation of MX1. Finally, a proper assessment of the expression of MX1 in a well-designed randomised clinical trial where patients are treated in a uniform fashion is recommended.

In conclusion, MX1 plays a role in BC associated with features of aggressive behaviour and is an independent prognostic marker associated with shorter survival. Its prognostic value is influenced by chemotherapy use; however, it is recommended for these results to be verified in a randomised clinical trial setting. Further functional studies in vitro and/or in vivo of the biological role of MX1 in BC cell lines are necessary to investigate its potential use as a therapeutic target in BC.

## Electronic supplementary material

Below is the link to the electronic supplementary material.

Supplementary file1 (DOCX 221 kb)
