# Supplementary material for: Myxovirus resistance 1 (MX1) is an independent predictor of poor outcome in invasive breast cancer
Source: Breast Cancer Res Treat. 2020 Apr 29;181(3):541–51. doi: 10.1007/s10549-020-05646-x (PMC7220876; doi:10.1007/s10549-020-05646-x)
Supplement: Supplementary file 1 — Supplementary file1 (DOCX 221 kb) [file 10549_2020_5646_MOESM1_ESM.docx]

**
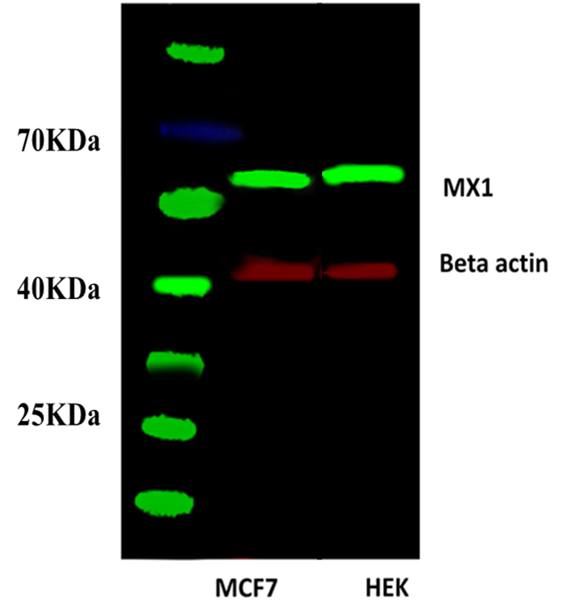
**

**Supplementary Figure (1)** Evaluation of MX1 primary antibody specificity by WB. This shows a single specific band at predicted size ( ~64KDa ).


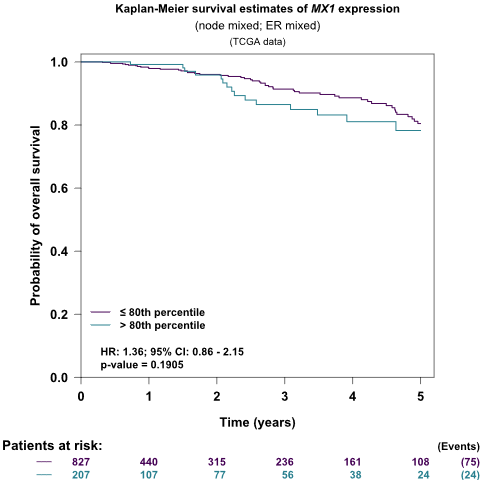


**Supplementary Figure (2)** Kaplan–Meier survival plots showing the association between MX1 RNA-seq data and patients’ outcome in publicly available dataset TCGA (n=1034) Breast Cancer Gene-Expression Miner v4.4 (bc-GenExMiner v4.4)


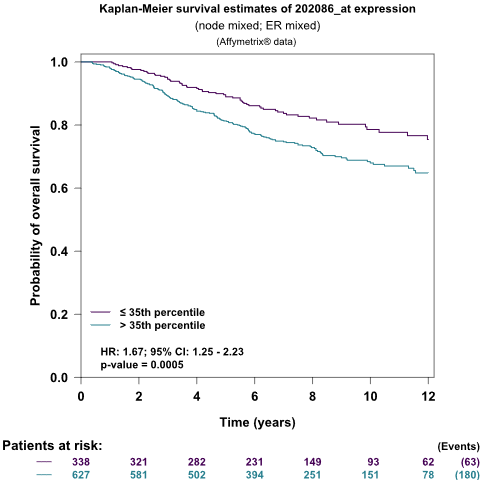


**Supplementary Figure (3)** Kaplan–Meier survival plots showing the association between MX1 DNA microarray data and patients’ outcome in publicly available datasets Affymetrix (n= 4904) Breast Cancer Gene-Expression Miner v4.4 (bc-GenExMiner v4.4)


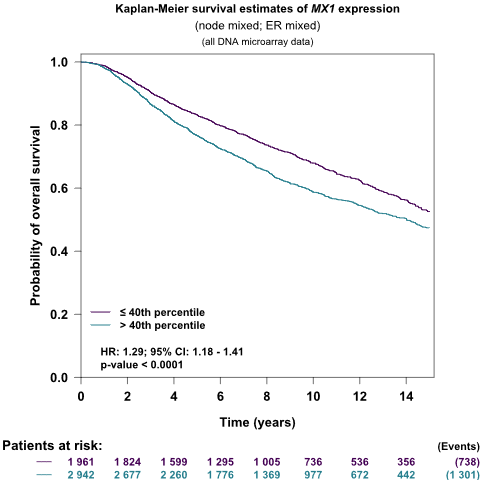


**Supplementary Figure (4)** Kaplan–Meier survival plots showing the association between all MX1 DNA microarray data and patients’ outcome in publicly available datasets (n= 10001) Breast Cancer Gene-Expression Miner v4.4 (bc-GenExMiner v4.4)


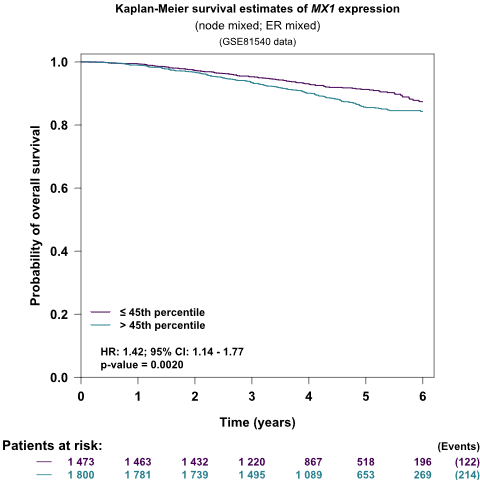


**Supplementary Figure (5)** Kaplan–Meier survival plots showing the association between MX1 RNA-seq data and patients’ outcome in publicly available dataset GSE81540 (n=3678) Breast Cancer Gene-Expression Miner v4.4 (bc-GenExMiner v4.4)


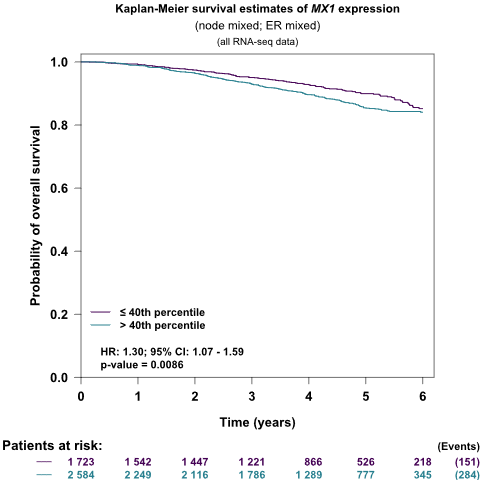


**Supplementary Figure (6)** Kaplan–Meier survival plots showing the association between all MX1 RNA-seq data and patients’ outcome in publicly available datasets (n= 4712) Breast Cancer Gene-Expression Miner v4.4 (bc-GenExMiner v4.4)
